# A pioneering health education strategy based on the PRECEDE-PROCEED model for women’s health challenges: a systematic review and logic framework

**DOI:** 10.1186/s12905-025-03883-0

**Published:** 2025-07-04

**Authors:** Khadijeh Khademi, Mohammad Hossein Kaveh

**Affiliations:** 1 Student Research Committee, Department of Health Promotion, School of Health, Shiraz University of Medical Sciences, Shiraz, 71536-75541 Iran; 2 Research Center for Health Sciences, Institute of Health, Department of Health Promotion, School of Health, Shiraz University of Medical Sciences, Shiraz, Iran

**Keywords:** Women's health, Health education, Quality of life, Health behavior, Social determinants of health

## Abstract

**Purpose:**

This review seeks to examine the effectiveness of PRECEDE-PROCEED model- based interventions on women’s behaviors, health, quality of life and determinants during health challenges as well as associations between their constructs. Additionally, it aims to propose a logical framework for enhancing women’s behaviors, health, quality of life and determinants during health challenges.

**Methods:**

In April 2025, three databases- PubMed, Scopus, and Science Direct- were searched. The search included words in the Title, Abstract, and Keywords without any time limitations. The search was filtered by Clinical Trial or Randomized Clinical Trial for PubMed, Article for Scopus, and Research articles for Science Direct. Studies conducted among women that utilized an educational intervention based on the PRECEDE-PROCEED model, were written in full-text English, and used validated quantitative health questionnaires were included in the analysis. Descriptive, cross-sectional designs as well as qualitative, longitudinal, or non-original studies were excluded from the review. The risk of bias in the included interventional studies was assessed using the Revised Cochrane risk-of-bias tool for randomized trials (RoB 2) and the Risk Of Bias In Nonrandomized Studies - of Interventions (ROBINS-I tool) which were specifically adapted for these study designs. The main objective of the interventional studies was to investigate the effectiveness of PRECEDE-PROCEED model-based interventions on women’s behaviors, health, quality of life and determinants during health challenges. A review was conducted based on the AMSTAR checklist, PRISMA checklist and PRISMA flow diagram. Data extraction was performed with the consensus of two authors, and a narrative synthesis approach was chosen for data synthesis, following the guidelines provided by the Centre for Reviews and Dissemination (CRD).

**Results:**

Seven interventional studies were included in the final analysis. Our findings revealed that all reviewed studies provided evidence of the effectiveness of a PRECEDE-PROCEED- based intervention for improving women’s behaviors, health, quality of life and determinants during health challenges. However, due to the absence of standardized measurement indicators to identify and compare the outcomes of various studies, there is a need to develop a conceptual framework that could enhance our understanding of women’s health challenges including childbirth, postpartum, menopause and genital infections. This framework aims to incorporate knowledge and positive attitudes towards healthy behaviors and health-related challenging conditions, as well as self-efficacy as important “predisposing factors.” It also considers social support and supportive policies as crucial “reinforcing factors.” Furthermore, it acknowledges resources, skills, services and their cost as necessary “enabling factors.” Lastly, it recognizes healthy behaviors and a favorable environment as “impacts,” alongside improved health and optimal quality of life as significant “outcomes” of these factors.

**Conclusions:**

The proposed conceptual framework could define a PRECEDE-PROCEED-based intervention as an effective “health education strategy” for addressing the most significant health challenges facing women.

## Introduction

Women still face many challenges in various aspects of health and well-being, which are often more pronounced in low-income and middle-income countries [1]. The World Health Organization (WHO) has identified ten top health issues and challenges worldwide including sexually transmitted infections, maternal health, aging, mental health, and more [2]. Women’s health issues exhibit similar patterns across various countries, including Iran [3–6]. Healthy People 2030 focuses on addressing various health challenges unique to women. Issues such as pregnancy, childbirth, and menopause can lead to specific health needs, required care, a decline in women’s safety, and an increase in the incidence of serious health problems and deaths [7]. Despite this, most studies on women’s health and wellbeing focus on a single stage within the life course, typically early childhood or reproductive years, despite the potentially progressive nature of health and well-being along the age continuum [1, 8]. Health problems can arise during pregnancy and childbirth, persist during the postpartum period, and can impact women’s physical, psychological, and social health [9]. Additionally, middle-aged and elderly women may experience a variety of disorders that can lead to death or hamper their quality of life [10]. Therefore, research that utilizes a life course approach and is informed by gender considerations aligns with a global agenda for women’s health [11]. All research findings should then be actively translated into care approaches for women worldwide [10].

Women seek continuity of care that takes into account their individual psychosocial circumstances and preferences [12]. On the other hand, providing high-quality healthcare at the lowest cost while maintaining accessibility is crucial for healthcare services [13]. Additionally, a comprehensive and strategic plan for women’s health can establish a long-term vision for improving health in the community [14]. This plan addresses questions such as where to focus, what actions should be taken, and how to achieve our goals [15, 16]. Therefore, it is essential from both clinical and public health perspectives to design and deliver comprehensive educational care interventions that are suitable for women [17, 18]. The PRECEDE-PROCEED model is a commonly used and valuable theoretical framework for planning, conducting, and evaluating comprehensive educational diagnosis and evaluation programs. It consists of Predisposing, Reinforcing, and Enabling Constructs as well as Policy, Regulatory, and Organizational Constructs in Educational and Environmental Development [19].

The PRECEDE-PROCEED model is a comprehensive framework for assessing health needs and designing, implementing, and evaluating health promotion and other public health programs to meet those needs. Therefore, all interventions that aim to promote behavioral change as a means of preventing diseases, improving health, and enhancing the quality of life in communities should be developed or evaluated via this model as a guide [20]. However, many studies don’t utilize all the constructs of this model in their interventions since comprehensive application requires significant resources, including time, funding, and personnel. This fact highlights the need for further research on how to make this approach more effective and explore strategies that are acceptable for protecting and improving health and quality of life during women’s challenges [21, 22]. Furthermore, the majority of studies on the PRECEDE-PROCEED model didn’t pay any attention to this important population specifically [23–25]. Despite recent advancements in understanding women’s health issues, factors and, promising care approaches, improving women’s health continues to be a challenge [10]. Additionally, it is crucial to acknowledge the health determinants experienced by women, as these can directly impact women’s healthcare needs. According to the principles of patient-centered care (PCC), healthcare and clinical decisions should be tailored to meet the needs, preferences, and values of patients [9]. Further research is needed to assist in the development of strategies that facilitate the process of implementing effective PRECEDE-PROCEED model- based interventions [19, 23].

Based on the background provided, the current review aims to achieve the following objectives: (1) assess evidence for the effectiveness of PRECEDE-PROCEED model- based interventions on women’s behaviors, health, quality of life and determinants during health challenges, (2) evaluate the evidence for associations between the PRECEDE-PROCEED constructs during women’s health challenges, and (3) propose a logical framework for enhancing women’s behaviors, health, quality of life and determinants during health challenges.

## Materials and methods

The present study utilized a systematic review based on the Preferred Reporting Items for Systematic Reviews and Meta-Analysis (PRISMA) [26] checklist. We chose three databases on the basis of recommendations from the PRISMA group paper, which suggests the need to search at least one database, and the Assessing the Methodological Quality of Systematic Reviews (AMSTAR) checklist, which recommends the search of at least two databases [27, 28]. Our search included the well-known databases PubMed, Scopus, and Science Direct, and we used the following search strategy (Table 1). Our investigation didn’t have any time limitations, as the number of articles wasn’t large.

**Table 1:** Search strategy for each database

**PubMed**	
(((PRECEDE-PROCEED[Title/Abstract]) **OR** (PRECEDE-PROCEED model[Title/Abstract]) **OR** (PRECEDE-PROCEED framework[Title/Abstract]))) **And** (women[Title/Abstract])	**Filters by**: Clinical Trial Randomized Clinical Trial
**Scopus**	
(((TITLE-ABS-KEY (precede proceed) **OR** TITLE-ABS-KEY (precede proceed model) **OR** TITLE-ABS-KEY (precede proceed framework))) **AND** (TITLE-ABS-KEY (women)	**Filters**: Article
**Science Direct**	
Title, abstract, keywords: (precede proceed **or** precede proceed model **or** precede proceed framework) **and** (women) **and** (clinical trial)	**Refine by**: Research articles

### Study criteria

The inclusion criteria for the studies consisted of five key factors: (1) the participation of women without any age limitation, (2) publication in full-text English, (3) interventional studies, (4) educational interventions based on the PRECEDE-PROCEED model without considering women’s health challenges, and (5) the use of validated health questionnaires to quantitatively assess the outcome. Descriptive or cross-sectional designs as well as qualitative, longitudinal, or non-original studies were excluded.

**Fig. 1:**
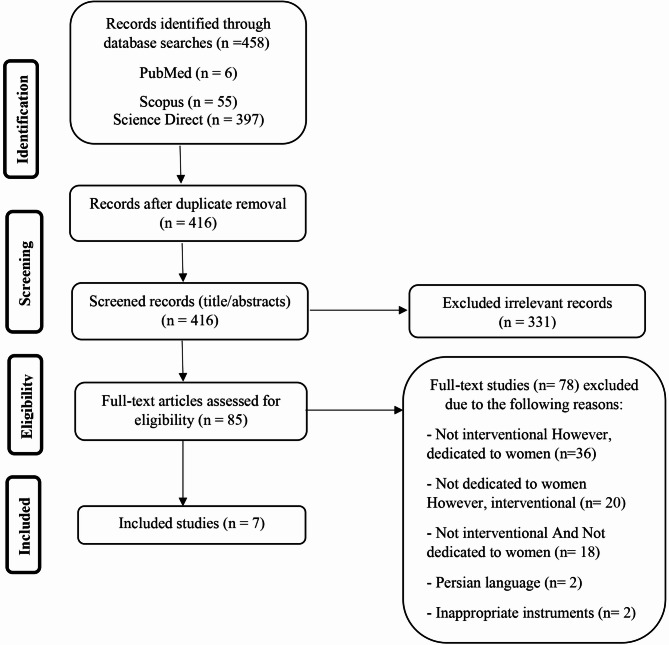
Study search and selection process

A total of 458 articles were initially identified in various databases in April 2025. This time frame aligned with the availability and collaboration schedule of the research team. The articles were then imported into EndNote 2025 (Bld 19000) software, with duplicates removed. Out of the 416 articles, 85 papers were selected for inclusion after being assessed for relevance by two independent reviewers who achieved consensus on which studies to include [26]. A total of 78 full-text studies were excluded for various reasons: 36 weren’t interventional studies but were dedicated to women, 20 were not dedicated to women but were interventional studies, and 18 were neither interventional studies nor dedicated to women. One had qualitative questionnaires, one used blood biomarkers as a measure of outcomes, and two were in the Persian language. Finally, 7 papers were reviewed. The study search and selection process are illustrated in Fig. 1.

### Risk of Bias in included studies

**Fig. 2:**
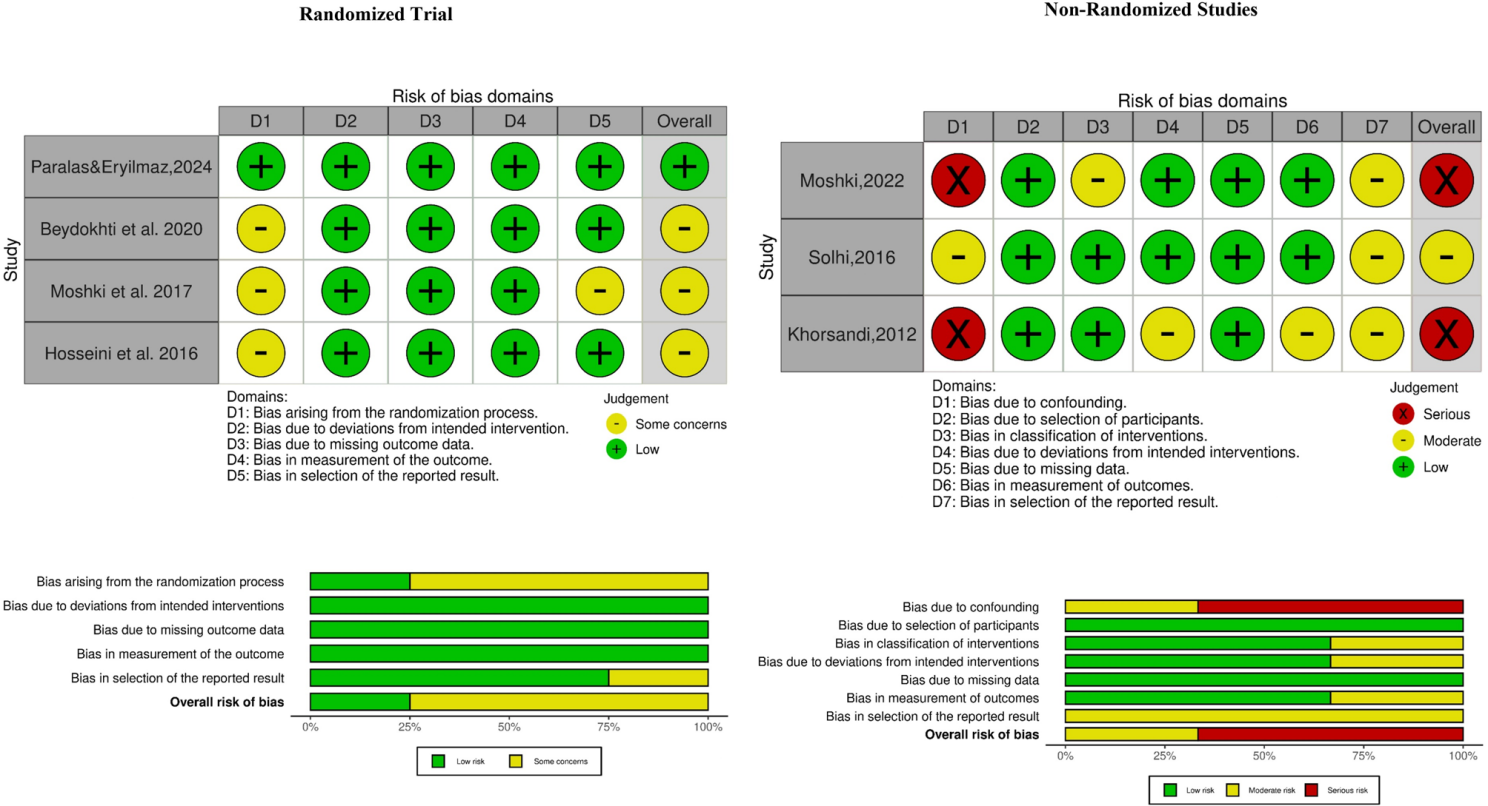
Summary of risk of bias; authors’ assessment of the risk of bias for each included study and each risk of bias item presented as percentages across all included studies

The Revised Cochrane risk-of-bias tool for randomized trials (RoB 2), version 22 August 2019, and the Risk Of Bias In Non-randomized Studies - of Interventions (ROBINS-I tool) were used to determine the risk of bias. One study was determined to have a low risk of bias, four had some concerns or moderate risk of bias, and two studies were assessed as having a serious risk of bias. Figure 2 presents the details of the risk-of-bias items separately for each article and summarizes all included studies.

### Data synthesis

A meta-analysis is often not feasible because of the presence of significant heterogeneity in quantitative indices or measurement tools. In such cases, a narrative approach to synthesis may be more appropriate and effective [29, 30]. Therefore, in the present study a narrative and deductive approach to synthesis was selected, following the steps outlined in the Centre for Reviews and Dissemination (CRD). These steps included developing a preliminary synthesis of the results of the included studies, exploring relationships in the data, and considering the robustness of the synthesis [31].

## Results

### Description of the studies

Seven studies were included in the final analysis. Two of them were quasi-experimental studies [32, 33], one was a clinical trial [34], one was a randomized clinical trial [35], and three were randomized controlled trials [36–38]. all of which took place in Asian nations. Only one study was conducted in Turkey [37], while the rest were conducted in Iran. The sample size varied from 48 [36] to 180 participants [33]. The participants included menopausal women [34, 38], pregnant women [32, 35], women-headed households [33], and those with gynecologic complications [36, 37]. The intervention duration varied from 4 weeks [35, 38] to 10 weeks [33]. The post-intervention test time range varied from immediately to 3 months. The characteristics of the studies are shown in Table 2.

**Table 2:** Characteristics of the included PRECEDE-PROCEED model-based RCT studies

Authors, Ref.	Country- year	Title	Women’s health challenges	Number of participants in analysis	Intervention duration	Time of post-intervention tests	PRECEDE-PROCEED evaluation		Type of study- Statistical analysis - Result
							Scales	Constructs	
Paralas& Eryilmaz, [37].	Turkey 2024	The effects of the PRECEDE-PROCEED model-based educational program on genital hygiene behaviors: A randomized controlled trial	Gynecological compliments	2 groups:120 I^1^: 60 C^2^: 60	5 weeks − 40 min/session	2 times: - Immediately − 4 weeks later	1- GHBI^3^ 2- Research-made questionnaire	-EA^4^: Behavior -PF^5^: Knowledge, Attitudes, Beliefs	**Randomized Controlled Trial**-Sample t & Cochran Q & Mc Nemar tests- Significant improvement in the mean score of Knowledge, Attitudes, and Behaviors related to genital hygiene within intervention group, which was significantly different from control group.
							**Objective: genital hygiene behaviors**		
Moshki et al. [34].	Iran 2022	Comparing the effect of education based on PRECEDE - PROCEED model in person-centered and supportive group methods on women’s psychological well-being in the menopausal period	Menopausal issues	3 groups:120 I^1^-person:40 I^1^-support:40 C^2^: 40	-	1 time	1- PWB^6^ 2- Research-made questionnaire	-SA^7^: Psychological well‑being -EA^4^: Behavior, Environment -PF^5^: Awareness, Attitudes, Self-efficacy -RF^8^: Social support -EF^9^: Educational resources, Skills	**Clinical Trial-** Kruskal-Wallis & Mann-Whitney U & Wilcoxon tests- Significant improvement in the mean score of Awareness, Attitudes, Self-efficacy, Environment-Behavior, Social support and Enablers of menopausal health within intervention groups, which were significantly different from control group. In addition, significant improvement in Psychological well‑being within supportive-intervention group, which was significantly different from control group.
							**Objective: psychological well‑being**		
Beydokhti et al. [35].	Iran 2020	Effect of educational- counseling program based on precede-proceed model during Pregnancy on postpartum depression	Maternal concerns	2 groups:130 I^1^: 60 C^2^: 70	4 weeks -60–90 min/session	1 time: - Immediately for GHQ^10^ and Research-made questionnaire − 4–6 weeks after child birth for EDPS^11^	1- EPDS^11^ 2- GHQ^10^ 3-Research-made questionnaire	-EA^4^: Health -PF^5^: Knowledge, Attitudes -RF^8^ -EF^9^	**Randomized Clinical Trial**- Independent t test & Linear regression- Significant differences were observed between the two groups in the mean score of Knowledge, Attitude, Reinforcing factors, Enabling factors and Depression prevalence. Attitude, Reinforcing and Enabling factors and General health were associated with post-partum depression.
							**Objective: postpartum depression**		
Moshki et al. [38].	Iran 2017	The effectiveness of a group-based educational program on the self-efficacy and self-acceptance of menopausal women: A randomized controlled trial	Menopausal issues	2 groups:80 I^1^: 40 C^2^: 40	4 weeks -120 min/session	1 time: - Immediately for Research-made questionnaire − 3 months later for SGSES^12^ and Self-acceptance subscale of PWB^6^	1- Self-acceptance subscale of PWB^6^ 2- SGSES^12^ 3- Research-made questionnaire	-SA^7^: Psychological well‑being -PF^5^: Self-efficacy -PF^5^: Knowledge, Attitudes -RF^8^: Social support -EF^9^: Educational resources, Skills	**Randomized Controlled Trial**- Paired t & Independent t & Wilcoxon & Mann-Whitney U tests- Between two groups were significant differences in the mean score of Predisposing, Reinforcing, and Enabling factors as well as self-efficacy and self-acceptance.
							**Objective: self-efficacy & self-acceptance**		
Hosseini et al. [36].	Iran 2016	Application of the PRECEDE model to improve sexual function among women with hysterectomy	Gynecological compliments	2 groups:48 I^1^: 24 C^2^: 24	5 weeks − 45–60 min/session	1 time: − 4 weeks later	1- FSFI^13^ 2- Research-made questionnaire	-EA^4^: Behavior -PF^5^: Knowledge, Attitudes -RF^8^ -EF^9^: Skills	**Randomized Controlled Trial-**Wilcoxon & Mann-Whitney U tests- The mean score of Knowledge, Attitude, Reinforcing factors, Enabling factors and Sexual function in experimental group were significantly higher than those in control group.
							**Objective: sexual function**		
Solhi et al. [33].	Iran 2016	A PRECEDE-PROCEED based educational intervention in quality of life of women-headed households in Iran	Gender inequality	2 groups:180 I^1^: 90 C^2^: 90	10 weeks − 40 min/session	2 times: − 1 month later − 3 months later	1- WHOQOL-BREF^14^ 2- Research-made questionnaire	-SA^7^: Health- related Quality of life -PF^5^: Knowledge, Attitudes, Behavioral causes -RF^8^ -EF^9^:	**Quasi-Experimental-** t-test & Mann-Whitney U & Repeated measurement analysis & Friedman test- After intervention, significant differences were observed between the mean scores of Predisposing factors, Enabling factors, Reinforcing factors, behavioral factors, and quality of life in the two groups.
							**Objective: quality of life**		
khorsandi et al. [32].	Iran 2012	The Effect of PRECEDE PROCEED Model Combined with the Health Belief Model and the Theory of Self-Efficacy to Increase Normal Delivery Among Nulliparous Women	Maternal concerns	2 groups:96 I^1^: 47 C^2^: 49	-	1 time: − 2 weeks later	1- Pregnancy outcome check list 2- CAQ^15^ 3- CBSEI^16^ 4- Self-recording labor-specific relaxation training check list 5 - Research-made questionnaire	-EA^4^: Behavior -PF^5^: Attitudes, Self-efficacy -EF^9^ -PF^5^: Knowledge, Attitudes -RF^8^: Social support, Policies -EF^9^: Services, Skills, Educational resources, Cost	**Quasi-Experimental-** Paired sample & independent t tests- Knowledge, Attitude, Self-efficacy, Enabling and Reinforcing factors were significantly increased after interventions in the case but not in control group. In addition, intervention was effective in increasing the rate of Normal Vaginal Delivery in case group.
							**Objective: normal delivery**		

^1^I: Intervention group, ^2^C: Control group, ^3^ GHBI: Genital Hygiene and Behaviors Inventory, ^4^EA: Epidemiological Assessment, ^5^PF: Predisposing factors, ^6^PWB: Ryff Psychological Well‑Being questionnaire, ^7^SA: Social Assessment, ^8^RF: Reinforcing factors, ^9^EF: Enabling factors, ^10^GHQ: General Health Questionnaire, ^11^EPDS: Edinburgh Postnatal Depression Scale, ^12^SGSES: Sherer’s General Self-Efficacy Scale, ^13^FSFI: Female Sexual Function Index, ^14^ WHOQOL-BREF: World Health Organization Quality of Life Questionnaire-BREF, ^15^CAQ: Harman’s Childbirth Attitudes Questionnaire, ^16^CBSEI: Lowe’s Child Birth Self-efficacy inventory

### Objective 1^a^: advancement of predisposing factors (educational & ecological domain)

All of the studies used a validated research-made questionnaire to assess predisposing factors. Two studies utilized Harman’s Childbirth Attitudes Questionnaire (CAQ), the Lowe’s Child Birth Self-Efficacy Inventory (CBSEI) [28], and the Sherer’s General Self-Efficacy Scale (SGSES) [38]. All of the studies evaluated the knowledge and attitude constructs. In comparison, only two studies assessed self-efficacy [32, 34], one study considered beliefs [37], and one measured behavioral causes [33] (see Table 2).

A PRECEDE-PROCEED model-based intervention could enhance knowledge and attitudes toward healthy behavior [32, 37], health challenges [34, 35, 38], self-efficacy [32, 34, 38], and behavioral causes [33] although beliefs do not [37].

### Objective 1^b^: reinforcement of reinforcing factors (educational & ecological domain)

All of the studies utilized a validated research-made questionnaire to assess reinforcing factors, except for one study [37] (refer to Table 2 for details). Three studies clearly noted that the constructs of social support (family, friends, and healthcare workers) [32, 34, 38] and supportive policies [32] were evaluated. An intervention based on the PRECEDE-PROCEED model could improve reinforcing factors [32–36, 38].

### Objective 1^c^: increment of enabling factors (educational & ecological domain)

All of the studies used a validated research-made questionnaire to measure enabling factors, with the exception of one study [37]. One study also evaluated enabling factors by utilizing a self-recording labor-specific relaxation training checklist [32] (refer to Table 2 for details). Four studies considered the constructs of access to educational or informational resources [32, 34, 38], necessary skills [32, 34, 36, 38], necessary services and cost [32] as enabling factors. A PRECEDE-PROCEED model-based intervention could promote enabling factors [32–36, 38].

### Objective 1^d^: enhancement of behavior or health or environment (epidemiological, behavioral & environmental domain)

Three studies utilized the Genital Hygiene and Behaviors Inventory (GHBI) [37], the Female Sexual Function Index (FSFI) [36], and the Pregnancy Outcome Checklist [32] to assess the construct of behavior. Only one study evaluated the behavior-environment factors via a validated research-made questionnaire [34]. Additionally, only one study assessed the construct of health via the General Health Questionnaire (GHQ) and the Edinburgh Postnatal Depression Scale (EPDS) [35] (refer to Table 2). The findings revealed that an intervention based on the PRECEDE-PROCEED model could improve behavior [32, 34, 36, 37], the environment [34] and health conditions [35].

### Objective 1^e^: improvement of quality of life or well-being (social domain)

The Ryff Psychological Well‑Being Questionnaire (PWB) and the World Health Organization Quality of Life Assessment-BREF (WHOQOL-BREF) were utilized in two studies [34, 38] and one study [33], respectively to evaluate the construct of health-related well-being (see Table 2). The results indicated that an intervention based on the PRECEDE-PROCEED model could improve quality of life [33] and psychosocial well-being [34, 38].

### Objective 2: association between constructs of the PRECEDE-PROCEED model

**Fig. 3:**
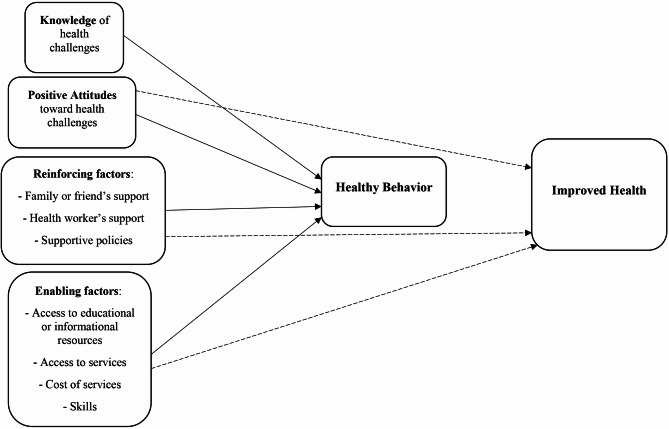
Associations between the constructs of the PRECEDE-PROCEED model in health challenges

One study revealed that attitudes toward health challenges, enabling factors, and reinforcing factors were contributing factors to health conditions [35]. Additionally, another study suggested that improving knowledge and attitudes toward health challenges; access to educational or informational resources and services; cost of services; and skills; and family, husband or health worker support and supportive policies can lead to healthy behaviors [32]. Figure 3 summarizes the studies, analyses, and syntheses pertaining to the associations among the constructs of the PRECEDE-PROCEED model in health-related challenging conditions.

## Discussion

In this study, we systematically reviewed interventional reports on PRECEDE-PROCEED model-based interventions for women facing health challenges. The review aimed to achieve three objectives: (1) assess the effectiveness of these interventions on women’s behaviors, health, quality of life and their determinants during health challenges, (2) evaluate the associations between PRECEDE-PROCEED constructs in the context of women’s health challenges, and (3) propose a logical framework for improving women’s behaviors, health, quality of life and their determinants during health challenges.

Regarding objective 1, our review revealed that interventions based on the PRECEDE-PROCEED model for women’s health challenges were significantly effective in improving behavior, health, quality of life and their determinants including predisposing, reinforcing and enabling factors. Other review studies also support our results, showing that interventions based on the PRECEDE-PROCEED model can enhance these health constructs in various conditions and populations. A systematic review and meta-analysis conducted by Kim et al. across various continents and countries, involving diverse populations and health challenges demonstrated that health program interventions using the PRECEDE-PROCEED model significantly improved behavior, health, quality of life, and their determinants [19]. Similarly, Tang et al., in their systematic review and meta-analysis conducted in China, Iran (Asia), Spain (Europe), and the USA (America), focusing on interventions based on the PRECEDE-PROCEED model, found that these interventions effectively reduced Glycated Hemoglobin A1c (HbA1c) levels and enhanced self-management among patients with type 2 diabetes [39]. The rationale behind this correlation is that the PRECEDE-PROCEED model can be served as a theoretical framework for health promotion interventions across diverse population groups with various contexts [19]. Therefore, this model is a valuable tool for all age groups and populations. Specifically, health determinants were effectively improved when interventions were implemented via this model. In the future, it will be beneficial to utilize the PRECEDE-PROCEED model when developing programs for managing health challenges and promoting health [19, 39].

Concerning objective 2, our systematic review indicated that attitudes, enabling factors, and reinforcing factors were contributing factors to health conditions. Additionally, improving knowledge and attitudes, access to enabling factors, and social support can lead to healthy behaviors. Predisposing factors, which include knowledge, beliefs, values, attitudes, reinforcing, and enabling factors, affect people’s motives for behavior change or adoption, resulting in better health [40, 41]. Similarly, Cho et al. conducted a real-world usability evaluation guided by the PRECEDE component for predisposing, enabling, and reinforcing factors influencing the use of a mobile-based HIV management app in New York city, USA, after a RCT. They discovered that knowledge, attitude, and self-efficacy for symptom management, design preference of illustrated strategies with videos, and user control were related to predisposing factors. Additionally, enabling factors were identified as skills and reinforcing factors were identified as social support and networking. All of these factors led to improvement in behavior that affected their quality of life [40]. Semblable, Borhani et al. conducted a quasi-experimental study to determine the effects of predisposing, reinforcing and enabling factors on self-care behaviors of patients with diabetes mellitus in Minoodasht city, Iran. They demonstrated that enabling factors, such as the ability to adopt healthy behaviors and educational classes as well as reinforcement factors like encouragement from health care staff, behavior results, and family advice improved knowledge, attitude, and behavior as predisposing factors [41]. These similarities can be explained by utilizing the constructs of the PRECEDE-PROCEED model in these studies [40, 41]. This model is intended to develop sustainable assessment and intervention programs, explore effective strategies to protect and enhance behavior, health, and quality of life, and facilitate the implementation of interventions and programs [19, 23, 42].

### Objective 3: A proposed logic framework for improving women’s behavior, health, quality of life and their determinants during health challenges

**Fig. 4:**
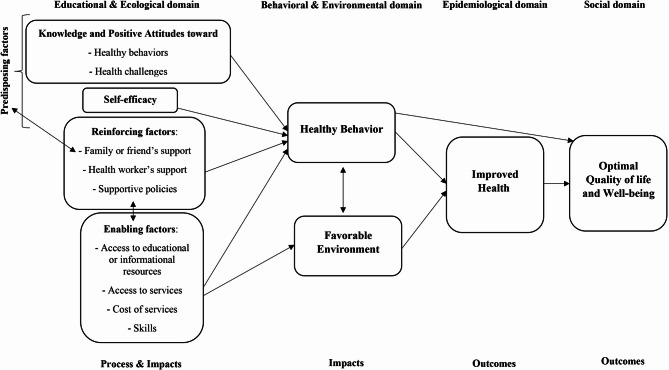
Proposed logic framework for improving women’s health challenges through the PRECEDE-PROCEED model- based health education strategy

PRECEDE-PROCEED model- based interventions were significantly effective in improving behaviors, health, quality of life and their determinants including predisposing, reinforcing and enabling factors during women’s health challenges. Therefore, these findings provide a foundation for developing and tailoring interventions and strategies to improve outcomes in these situations [19, 23]. The major components of our proposed logic framework are depicted in Fig. 4 and described below.

In the logic framework, predisposing factors are linked to knowledge and positive attitudes toward healthy behavior [32, 37] health challenges [34, 35, 38], and self-efficacy [32, 34, 38]. Additionally, reinforcing factors include support from family, friends or health workers [32, 34, 38] and supportive policies [32]. Enabling factors include access to educational or informational resources [32, 34, 38], necessary skills [32, 34, 36, 38], necessary services and the cost of services [32]. Finally, the impacts and outcomes of this framework are healthy behaviors [32, 34, 36, 37], a favorable environment [34], improved health [35] and optimal quality of life or well-being [33, 34, 38]. The use of implementation science frameworks and methods will help policy makers more rigorously and systematically evaluate and develop best practices for implementing health services [43]. Additionally, the conceptual model should be used in research, health promotion, public health practice, and quality improvement programs to improve methodological rigor and, consequently, health equity [39]. The key message for practice is that the application of implementation models from the inception of intervention can help researchers and practitioners leverage known facilitators and mitigate the impact of barriers [23].

The present systematic review is limited by its focus on English-language publications of the included studies. Additionally, a meta-analysis was not conducted due to shortcomings in quantitative indices and varying measuring tools. The novel contribution of this study is the development of a conceptual framework that offers insights into the design of programs via the PRECEDE-PROCEED model. We recommend the use of standard protocols for designing, implementing, and evaluating interventions to facilitate comparisons in systematic reviews and meta-analyses studies. Our proposed logic framework can be tested as a guiding framework in intervention design.

## Conclusions

In conclusion, this systematic review highlights the effectiveness of PRECEDE-PROCEED model- based interventions in improving behavior, health, quality of life and their determinants including predisposing, reinforcing and enabling factors during women’s health challenges. The conceptual model should be used in research, health promotion, public health practice, and quality improvement programs to improve methodological rigor and, consequently, health equity.

## Data Availability

The datasets used and/or analyzed during the current study are available from the corresponding author upon reasonable request.
